# Longitudinal changes in global structural brain connectivity and cognitive performance in former hospitalized COVID-19 survivors: an exploratory study

**DOI:** 10.1007/s00221-023-06545-5

**Published:** 2023-01-28

**Authors:** B. Tassignon, A. Radwan, J. Blommaert, L. Stas, S. D. Allard, F. De Ridder, E. De Waele, L. C. Bulnes, N. Hoornaert, P. Lacor, E. Lathouwers, R. Mertens, M. Naeyaert, H. Raeymaekers, L. Seyler, A. M. Van Binst, L. Van Imschoot, L. Van Liedekerke, J. Van Schependom, P. Van Schuerbeek, M. Vandekerckhove, R. Meeusen, S. Sunaert, G. Nagels, J. De Mey, K. De Pauw

**Affiliations:** 1 Human Physiology and Sports Physiotherapy Research Group, Vrije Universiteit Brussel, Brussels, Belgium; 2 BruBotics, Vrije Universiteit Brussel, Brussels, Belgium; 3 Strategic Research Program ‘Exercise and the Brain in Health & Disease: The Added Value of Human-Centered Robotics’, Vrije Universiteit Brussel, Brussels, Belgium; 4 Department of Radiology and Magnetic Resonance, UZ Brussel, Jette, Belgium; 5 Artificial Intelligence and Modelling in Clinical Science, Vrije Universiteit Brussel, Brussels, Belgium; 6 Department of Imaging and Pathology, Translational MRI, KU Leuven, Leuven, Belgium; 7 Department of Radiology, UZ Leuven, Leuven, Belgium; 8 Department of Oncology, KU Leuven, Leuven, Belgium; 9 Biostatistics and Medical Informatics Research Group, Department of Public Health, Faculty of Medicine and Pharmacy, Vrije Universiteit Brussel, Brussels, Belgium; 10 Interfaculty Center for Data Processing and Statistics, Core Facility Statistics and Methodology, Vrije Universiteit Brussel, Brussels, Belgium; 11 Department of Electronics and Informatics (ETRO), Vrije Universiteit Brussel, Brussels, Belgium; 12 Brain, Body and Cognition Research Group, Faculty of Psychology, Vrije Universiteit Brussel, Brussels, Belgium; 13 Infectious Diseases Unit, Department of Internal Medicine, UZ Brussel, Jette, Belgium; 14 Intensive Care Unit, UZ Brussel, Jette, Belgium

**Keywords:** Magnetic resonance imaging, Recovery, SARS-CoV-2

## Abstract

**Background:**

Long-term sequelae of COVID-19 can result in reduced functionality of the central nervous system and substandard quality of life. Gaining insight into the recovery trajectory of admitted COVID-19 patients on their cognitive performance and global structural brain connectivity may allow a better understanding of the diseases' relevance.

**Objectives:**

To assess whole-brain structural connectivity in former non-intensive-care unit (ICU)- and ICU-admitted COVID-19 survivors over 2 months following hospital discharge and correlate structural connectivity measures to cognitive performance.

**Methods:**

Participants underwent Magnetic Resonance Imaging brain scans and a cognitive test battery after hospital discharge to evaluate structural connectivity and cognitive performance. Multilevel models were constructed for each graph measure and cognitive test, assessing the groups' influence, time since discharge, and interactions. Linear regression models estimated whether the graph measurements affected cognitive measures and whether they differed between ICU and non-ICU patients.

**Results:**

Six former ICU and six non-ICU patients completed the study. Across the various graph measures, the characteristic path length decreased over time (*β* = 0.97, *p* = 0.006). We detected no group-level effects (*β* = 1.07, *p* = 0.442) nor interaction effects (*β* = 1.02, *p* = 0.220). Cognitive performance improved for both non-ICU and ICU COVID-19 survivors on four out of seven cognitive tests 2 months later (*p* < 0.05).

**Conclusion:**

Adverse effects of COVID-19 on brain functioning and structure abate over time. These results should be supported by future research including larger sample sizes, matched control groups of healthy non-infected individuals, and more extended follow-up periods**.**

## Introduction

More than 350 million cases of coronavirus disease 2019 (COVID-19) have been reported worldwide as of February 2022 and over 5.6 million people died due to COVID-19 (European Centre for Disease Prevention and Control 2022). Severe acute respiratory syndrome coronavirus 2 (SARS-CoV-2) is the causative agent of COVID-19 (Gorbalenya et al. 2020). Despite respiratory distress being the most characteristic symptom of COVID-19, SARS-CoV-2 has neuroinvasive and neurotropic capabilities which may result in neuropathological consequences (Fotuhi et al. 2020; Iadecola et al. 2020; Montalvan et al. 2020; Song et al. 2021; Yachou et al. 2020). Even mild COVID-19 is often suggested to result in long-term consequences on the functionality of the central nervous system and on the quality of life (Duong 2021; Lu et al. 2020; Frontera et al. 2021b).

Neurological manifestations are estimated to be present in one third of COVID-19 patients (Mao et al. 2020). Both subjective neurological symptoms as well as objective neurological signs are frequently reported (Liguori et al. 2020; Meppiel et al. 2021; Frontera et al. 2021a; Luigetti et al. 2020; Bahranifard et al. 2021). A large longitudinal multimodal magnetic resonance imaging (MRI) study revealed the detrimental impact of COVID-19 on regional gray matter and whole-brain volume when comparing brain scans acquired from individuals before and after SARS-CoV-2 infection with brain scans from a healthy control group (Douaud et al. 2022). An MRI-based follow-up study of COVID-19 patients using diffusion tensor imaging (DTI) found at the 3-month interval an overall decrease in mean diffusivity, axial diffusivity, and radial diffusivity in combination with an increase in fractional anisotropy compared to participants without COVID-19. Global mean diffusivity was negatively correlated with memory loss (Lu et al. 2020). Contrasting observations were made by Yang et al. (2021). Using DTI, in combination with a local and whole-brain graph theory analysis in recovered COVID-19 patients, decreased fractional anisotropy and increased mean- and radial diffusivity values in widespread brain regions were observed as well as significantly lower global efficiency and longer characteristic path length. Also, a less nodal local efficiency in the superior occipital gyrus was detected (Yang et al. 2021). Yet, both studies reported the observed white matter changes as unfavorable when comparing recovered COVID-19 patients to healthy controls. Further research is needed to investigate whether the deleterious impact of COVID-19 on white matter microstructure is partially reversible, or whether these unfavorable changes will persist both in the short and long term. Therefore, in this longitudinal study, we will use graph theory with CSD tractography-based structural connectomes to investigate changes in structural brain reorganization COVID-19 hospitalization.

Related to these adverse brain changes, considerable cognitive deficits were observed in the subacute stage of COVID-19 (Hosp et al. 2021) and in people who recovered from COVID-19 (Hampshire et al. 2021; Douaud et al. 2022). Compromised cognitive functioning may lead to reduced work-related and functional outcomes for individuals recovering from COVID-19 with a potentially greater risk of cognitive decline and dementia in later life (Cothran et al. 2020; de Erausquin et al. 2021; Del Brutto et al. 2021b). The review by Hopkins and Jackson (2006) found that current critical illness research indicates that cognitive sequelae are commonly present after Intensive Care Unit (ICU) treatment and discharge. These cognitive impairments may be permanent and are associated with impairments in activities of daily living, lower quality of life, and inability to return to work (Hopkins and Jackson 2006; Tasker and Menon 2016). However, there are some indications that cognitive functioning and brain structure might recover on the longer term. The prospective study of Kanberg and colleagues (Kanberg et al. 2021) noted normalization of plasma levels of central nervous system injury biomarkers in COVID-19 patients after 6 months regardless of previous disease severity. Another prospective study found a slow but evident recovery in neocortical dysfunction and cognitive impairments in eight chronic COVID-19 patients (Blazhenets et al. 2021). Even though these results are encouraging, longitudinal cognitive and neuroimaging studies are warranted to map recovery trajectories and invest the neural basis of cognitive deficits in SARS-CoV-2 survivors.

Gaining insight in the recovery trajectory of admitted COVID-19 patients on cognitive performance and global structural brain connectivity may further contribute to better understand the potential pathological relevance of the infection at the physiological, structural, and cognitive level. Moreover, to the best of our knowledge, it is currently unknown how structural brain connectivity and cognitive performance evolve in the first month following discharge from the ICU or the non-ICU COVID-19 ward. The current study assesses whole-brain structural connectivity in former non-ICU- and ICU-admitted COVID-19 survivors. For a period up to 2 months after hospital discharge, recovered patients underwent several exams in order to determine clinical MRI data, graph theoretical measures derived from diffusion MRI (dMRI) tractography and cognitive performance by means of standardized cognitive test battery assessing different cognitive domains. We hypothesize that structural brain connectivity improves in both groups over a period of 2 months after hospital discharge. We also hypothesize that cognitive performance in terms of reaction time over different cognitive tasks improves in both groups over a period of 2 months after hospital discharge. Moreover, we hypothesize that structural brain connectivity and cognitive performance in terms of reaction time would be worse in former ICU-treated COVID-19 survivors compared to non-ICU-treated COVID-19 survivors. Furthermore, we aim to link these structural brain connectivity measures to cognitive performance.

## Methods

### Study design, standard protocol approvals, registrations, and patient consents

This was a prospective observational single-center study performed at the University Hospital Brussels (UZ Brussel, Jette, Belgium). The study was approved by the Medical Ethics Committee of the UZ Brussel (B.U.N. 1432020000338). The study protocol and procedures were registered and released on ClinicalTrials.gov Protocol Registration and Result System (NCT04726176) and are in accordance with the Declaration of Helsinki. Before participating in the study, all participants provided written informed consent and could ask further questions concerning the study.

### Patient cohort and study protocol

We planned to enroll 20 patients who were admitted at the UZ Brussel with clinical signs of COVID-19 pneumonia to undergo a Magnetic Resonance Imaging (MRI) brain scan and a cognitive test battery twice. The first time was at 1–2 months after hospital discharge, and a second time at 3–4 months after hospital discharge. All tests and measurements were conducted at the department of Radiology-Magnetic Resonance (UZ Brussel). Only patients with a positive reverse transcriptase-polymerase chain reaction test (RT-PCR) were included in this study. Both patients admitted on the Intensive Care Unit (ICU) and on the regular COVID-19 ward (non-ICU) were eligible for inclusion.

Patient recruitment was performed by a radiology resident in collaboration with the department of infectious diseases. A list of all hospitalized COVID-19 patients at the UZ Brussel was created by the Intensive Care Unit and Infectiology Department. These patients were contacted personally by phone by an infectious disease specialist or were asked during a follow-up consultation whether they were willing to participate in this study. Patients willing to participate were then contacted by the radiology resident to further explain the study protocol.

### Magnetic resonance imaging of the brain

Table 1 shows the detailed information of each brain imaging technique. Brain MRI in supine position was conducted on a 3 Tesla MRI Ingenia scanner (Philips Medical Systems, Best, The Netherlands). The protocol contained axial 2D-T2 weighted images, 3D T1-weighted spin-echo images, and diffusion-weighted imaging (DWI). A diffusion tensor imaging (DTI) sequence was also performed (48 directions at a *b *value of 3000 s/mm^2^). After administration of a gadolinium-based contrast agent (Dotarem^®^, Guerbet), susceptibility-weighted imaging (SWI), 3D T1-weighted spin-echo images, and a late fat-saturated 3D-FLAIR were acquired.

**Table 1:** Characteristics of the different brain imaging techniques

	2D T2w TSE	3D T1	3D SWI	3D-FLAIR	DWI (3 directions)	HARDI (48 directions)
FoV (RL, AP, FH) (mm)	183 × 230 × 149	160 × 230 × 230	178 × 230 × 130	159 × 230 × 230	230 × 230 × 149	224 × 224 × 120
Acquisition resolution (mm)	0.575 × 0.62	1 × 1	0.8 × 0.8	1.2 × 1.2	1.5 × 1.89	2 × 2
Slice thickness (+ gap) (mm)	4 + 1	1	2	1.2	4 + 1	2 + 0
Reconstruction resolution (mm)	0.45 × 0.45 × 4.0	0.53 × 0.53 × 0.50	0.3 × 0.3 × 1.0	0.6 × 0.6 × 0.6	0.9 × 0.9 × 4.0	2.0 × 2.0 × 2.0
BW/pix (Hz/pixel)	242.2	434.5	255.3	1112.8	20.0	16.8
BW in EPI freq dir (Hz/pixel)					1448.7	1872.0
Turbo field echoes		220		182		
Turbo spin echoes	19					
Number of echoes			4			
Flip angle (degrees)		8	17			
Echo time (ms)	80	2.3	7.2	337	111	127
Echo spacing (ms)	8		6.2	3.4		
Repetition time (ms)	3432	5.2	31	4800	4561	5164
*b*-factor (s/mm^2^)					1000	3000
Acceleration technique	CSENSE	CSENSE	CSENSE	CSENSE	SENSE	SENSE + MULTIBAND
Acceleration factor	2	1.3	7	5.5	2	1.3 + 3 × multiband
Acquisition time (min:s)	00:55	03:19	01:14	04:05	00:55	04:24

On the second visit, a follow-up MRI examination for possible brain disease was done on the same MRI scanner, consisting of axial 2D-T2-weighted images, 3D T1-weighted spin-echo images, fat-saturated 3D- FLAIR, SWI, and DWI, followed by the DTI sequence. No gadolinium-based contrast agent was administrated for follow-up.

### Primary outcome measures

#### Structural brain connectivity

1. Image pre-processing and analysis

Diffusion-weighted images were pre-processed using the KU Leuven neuroimaging suite (KUL_NIS) (Sunaert and Radwan 2021), which relies on MRTrix3 (v. 3.0.3) (Tournier et al. 2019), FSL (v. 6.0) (Jenkinson et al. 2012), ANTs (v. 2.3.1) (Avants et al. 2011), and Synb0DisCo(Schilling et al. 2019), to correct for imaging noise, Gibb’s ringing, Eddy and head motion artifacts, echo-planar imaging (EPI) distortion, and image intensity bias. Quantitative quality assessment of the pre-processed diffusion images was done in FSL (Bastiani et al. 2019) and one dataset had to be excluded due to large imaging artifact. 3D T1-weighted images were processed using the FreeSurfer (v. 6.0.0) recon-all pipeline (Desikan et al. 2006, Fischl 2012). Constrained spherical deconvolution (CSD) (Tournier et al. 2007) was used to calculate a group-averaged white matter response function, which was used to generate white matter fiber orientation distribution maps for all subjects. Probabilistic tractography with second-order integration over orientation distributions (iFOD2) (Tournier et al. 2010) anatomically constrained tractography (ACT) (Smith et al. 2012) was used to generate whole-brain tractograms with 10 million streamlines for each subject, and spherical-deconvolution informed filtering (SIFT2) (Smith et al. 2013, 2015) of tractograms was used to minimize spurious streamlines.

2. Structural connectome construction

We used the whole-brain tractograms and the Desikan–Killinay parcellations (Desikan et al. 2006) generated by FreeSurfer to construct structural connectomes in MRTrix3. Each of the 84 anatomical brain regions defined in the parcellation maps was represented by a node in the resulting network. The edges in these networks represent the white matter connections between each pair of brain regions. Resulting connectomes are weighted networks, where each edge is weighted by the sum of streamline-weights derived from SIFT2 for the white matter connections between each pair of nodes.

3. Network analysis and graph theory measures’ calculation

Global graph theory measures were calculated in the Brain connectivity toolbox (v2019-03-03) and in-house MATLAB code (v2020a). Characteristic path length and global efficiency were calculated using Dijkstra’s algorithm, with the connection-length matrix defined by the inverse edge weights. Clustering coefficient and local efficiency measures were calculated as recommended by Wang and colleagues (Wang et al. 2017). For each connectome 100 random graphs were calculated using random permutation, while keeping connectome symmetry and excluding graphs with disconnected nodes. Each graph metric was normalized by dividing the metric by the median metric of the random graphs for that subject.

#### Cognitive performance

The computerized cognitive test battery "Cognition" (Joggle^®^ Research, Seattle, WA, USA) was conducted using an iPad. The average duration of the cognitive test battery is 18 min. This cognitive test battery is sensitive to multiple domains at high-level cognitive performance and has been proven to engage specific brain regions evidenced by functional neuroimaging (Basner et al. 2015). It consists of the motor praxis test (measure of sensorimotor speed), visual object learning test (measure of spatial learning and memory), abstract matching (measure of abstraction), line orientation test (measure of spatial orientation), digit symbol substitution test (measure of complex scanning and visual tracking), balloon analogue risk test (measure of risk decision-making), NBACK (measure of working memory), and psychomotor vigilance test (measure of vigilant attention). Before actually performing each cognitive test, participants practiced each cognitive test once to mitigate learning effects. Detailed description of each cognitive test can be found in the work of Basner and colleagues (Basner et al. 2015). Median reaction time was the main outcome measure of interest for every cognitive test.

#### Correlation structural brain connectivity and cognitive performance

Only observations from the first time point were used. Because of the low sample size, a step-wise model-building technique was used. The Akaike Information Criterion (AIC) is used for performing the model selection. The AIC estimates the quality of each model, relative to each of the other models. The smaller the AIC, the better the fit. The analyses were performed using the step()-function from the MASS package using both forward and backward selection.

### Secondary outcome measures

#### Anatomical brain imaging

To detect abnormal brain patterns, we segmented a T1-weighted image into white matter, gray matter, and cerebrospinal fluid using the Icometrix icobrain pipeline (version 3.1) (Jain et al. 2015). FLAIR white matter hyperintensities were detected and included in the white matter segmentation. After performing skull stripping and bias correction, we segmented the T1-weighted image using a probabilistic image intensity model, and non-rigidly propagated tissue priors from an MNI atlas (Mazziotta et al. 1995). We obtained lesion segmentation by iterating a loop until convergence that comprised T1-weighted image segmentation, identifying intensity outliers on the FLAIR image, and filling the lesions on the T1-weighted image (Jain et al. 2015; Smeets et al. 2016). T1 hypointensities, commonly known as black holes, were obtained as a sub-segmentation of the FLAIR lesions. There was some contention over the sensitivity of 3D T1 sequencing in identifying T1 hypointensities. A 3D sequencing often detects more albeit less severe hypointensities (Lapucci et al. 2020). Icobrain refines the primary tissue segmentation to obtain cortical gray matter and thalami sub-segmentations (Jorge Cardoso et al. 2013). We normalized brain volumes for head size except for lesion load and black hole volume. To conclude, the icobrain pipeline allowed the estimation of whole-brain white matter, deep and cortical gray matter, lesion burden, and black hole and thalamic volumes to detect abnormality patterns.

### Statistical analyses

All statistical analyses were performed using R (version 4.1.2; R Core Team 2021). A p value below 0.05 was considered to be statistically significant. Multilevel models were fitted using the lme4 (Bates et al. 2015) and lmerTest (Kuznetsova et al. 2017) package, allowing to take into account the clustering in the data and all available observations. First, multilevel models are constructed for each graph measure separately testing the role of group (ICU = 0, non-ICU = 1), time (at hospital discharge = 0, follow-up = 1), and their interaction, simultaneously, while allowing random intercepts for the participants. Next, multilevel models are constructed for each cognitive test, estimating the role of the same predictor variables. When data were normally distributed, general multilevel models were used. For nonnormally distributed data, generalized multilevel models were explored and applied. That is, multilevel models using different distributions and link functions were fitted, and the best model fit was found by means of Bayesian information criterion (BIC) values and—in case of doubt—formal likelihood ratio tests were performed. An overview with the different models together with their properties can be found in Table 3. For the last hypothesis, using linear regression models, we tested if the graph measures have an effect on the cognitive measures right after hospital discharge and if there is significant difference between ICU and non-ICU patients. Due to the small sample size and the large number of parameters of interest, a step-wise model-building approach was chosen to avoid an inflation of the Type I error rate and to obtain the most parsimonious model per outcome variable. This technique aims to avoid overfitting the data and results in smaller standard errors. These analyses were performed using the step()-function from the MASS package using both forward and backward selection (Venables and Ripley 2002). To avoid multicollinearity, predictor variables were grand mean centered.

## Results

### Participant characteristics

Participant characteristics can be retrieved in Table 2. A total of 20 COVID-19 patients were included in this study. One patient dropped out due to personal reasons; thus, data of the remaining 19 patients were analyzed. All included patients had a positive RT-PCR result at the time of admission. Six patients (32%) were hospitalized in the intensive-care unit due to respiratory distress and/or clinical deterioration. Two ICU patients required mechanical ventilation. The other 13 patients (68%) were hospitalized at the regular COVID-19 ward and did not need intensive care.

**Table 2:** Patient characteristics

	ICU	NICU
Mean age ± SD (years)	51 ± 13	59 ± 10
Age range (years)	21–64	36–76
Sex (M/F)	7/2	8/2
BMI (kg/m^2^)	28 ± 4	27 ± 4
Smoking (%, *n*)	22%, *n* = 2	0%, *n* = 0
ICU patients/non-ICU patients	9	11
Mean length of hospital stay ± SD (days)	18 ± 8	8 ± 3
Neurological symptoms at the time of hospital admission		
Headache	44%, *n* = 4	36%, *n* = 4
Concentration problems	22%, *n* = 2	45%, *n* = 5
Fatigue	22%, *n* = 2	27%, *n* = 3
Agitation	11%, *n* = 1	27%, *n* = 3
Memory disorders	11%, *n* = 1	27%, *n* = 3
Delirium	11%, *n* = 1	18%, *n* = 2
Decreased consciousness	0%, *n* = 0	18%, *n* = 2
Vertigo	0%, *n* = 0	9%, *n* = 1
Neurological symptoms at the time of first experimental session		
Concentration problems	22%, *n* = 2	36%, *n* = 4
Fatigue	0%, *n* = 0	27%, *n* = 3
Memory disorders	11%, *n* = 1	27%, *n* = 3
Comorbidities		
Type 2 diabetes mellitus	67%, *n* = 6	9%, *n* = 1
Overweight and obesity	78%, *n* = 7	64%, *n* = 7
Arterial hypertension	22%, *n* = 2	27%, *n* = 3
Dyslipidaemia	33%, *n* = 3	9%, *n* = 1
Migraine	0%, *n* = 0	9%, *n* = 1
Alcohol dependence	0%, *n* = 0	9%, *n* = 1
Chronic obstructive pulmonary disease (COPD)	11%, *n* = 1	9%, *n* = 1
Asthma	0%, *n* = 0	9%, *n* = 1
Fibromyalgia	11%, *n* = 1	0%, *n* = 0
Ulcerative colitis	0%, *n* = 0	9%, *n* = 1
Hypothyroidism	0%, *n* = 0	9%, *n* = 1

Only 6 patients (30%) had resumed work as before at inclusion (about 1–2 months after hospital discharge). Three patients (15%) had to work part-time and 6 patients were still unable to work due to disability (30%). Five patients (25%) did not work before their admission due to unemployment or retirement.

On the first visit, 19 out of 20 patients underwent the whole experimental session (cognitive tests and MRI brain scan). One patient refused to do the MRI brain scan and only did the cognitive tests. Ten out of 20 patients did not report any of the previously mentioned neurological symptoms upon their visit.

On the second visit (2 months later), 12 of the 20 patients (of which 6 ICU and 6 non-ICU patients) returned to perform the follow-up cognitive testing and MRI scan. The main reasons for drop-out were due to illness, lack of time, or claustrophobia experienced during the previous MRI scan.

### Anatomical MRI findings

None of the patients had an objectifiable neurological deficit. Also, we detected no COVID-19-related brain lesions such as cerebral microhemorrhages, acute spontaneous intracranial hemorrhage, acute to subacute infarcts, and encephalitis or encephalopathy (Lersy et al. 2021; Kremer et al. 2020a, b). Yet, there was one patient that had an old right frontal periventricular white matter lesion. The Icometrix reports indicated that none of the patients had abnormal age-related brain atrophy.

### Structural brain connectivity

**Fig. 1:**
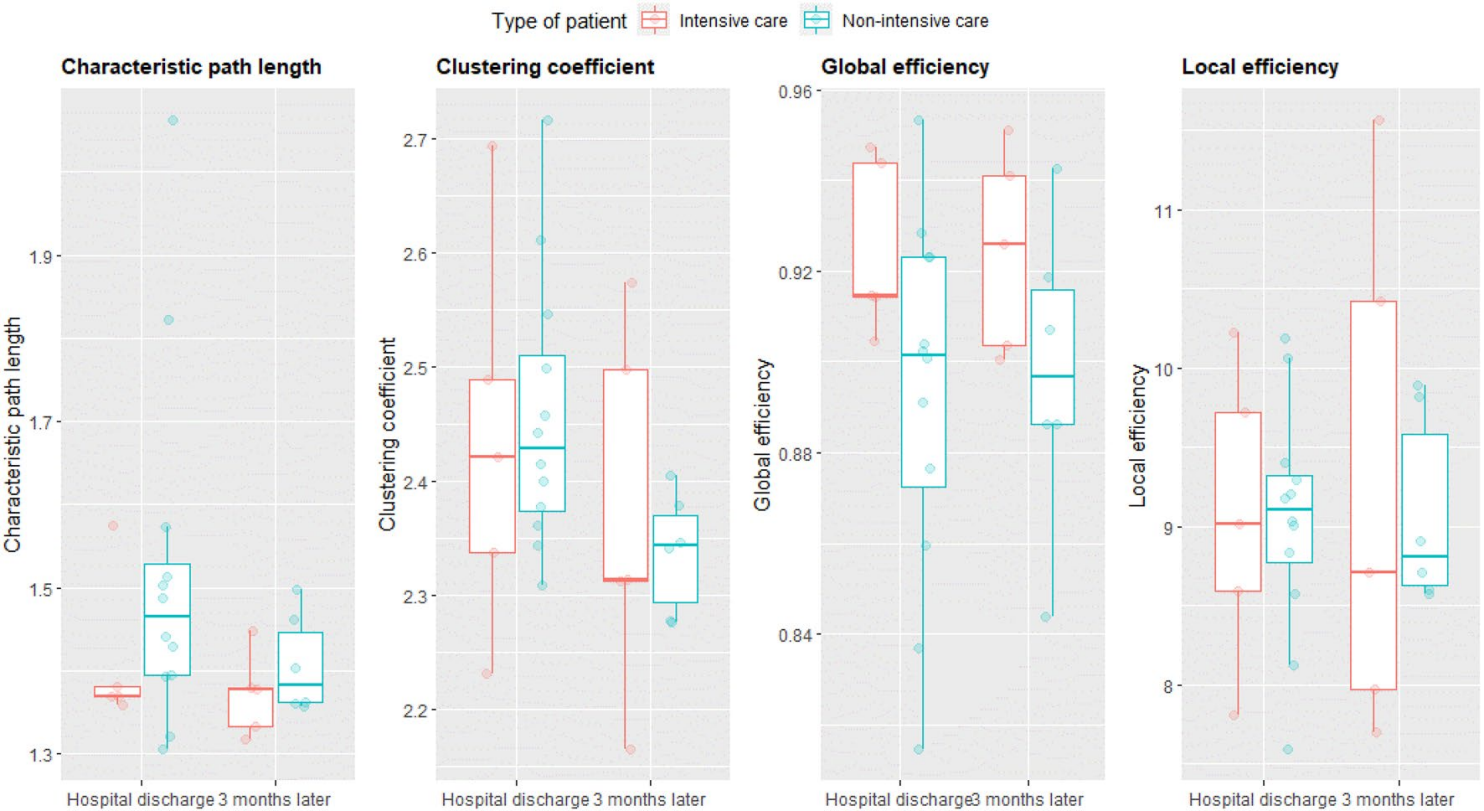
Graph theory measures of ICU (red) and non-ICU (blue) care patients at hospital discharge and 2 months later. Dots represent individual patient scores

Figure 1 depicts the normalized whole-brain structural graph metrics of ICU and non-ICU patients at hospital discharge and 2 months later. For each metric, Table 3 shows the final models, their estimates, and corresponding significance level, and BIC and ICC values.

**Table 3:** Results of the multilevel models for the effect of time, group, and their interactions on different graph measures: estimated regression coefficients, estimated variance components, and model information

Outcome	Fixed effects *B*(SE)				Random effects		Model Info		
	Intercept	Time(2)	Group(nicu)	Time(2) × Group(nicu)	$\sigma_{{{\text{pat}}}}^{2}$	$\sigma_{{{\text{res}}}}^{2}$	Distribution (link)	ICC	BIC
Characteristic path length	0.34 (0.07)***	– 0.03 (0.01)**	0.064 (0.08)	0.02 (0.01)	0.09	0.05	Gaussian (link = log)	0.76	– 66.51
Cluster coefficient	2.43 (0.05)***	– 0.06 (0.04)	0.002 (0.07)	– 0.01 (0.06)	0.009	0.005	Gaussian (link = identity)		
Global efficiency	– 0.08 (0.03)**	– 0.0006 (0.004)	– 0.04 (0.03)	– 0.004 (0.005)	0.0009	0.0001	Gaussian (link = log)	– 149.13	
Local efficiency	9.07 (0.43)***	0.20 (0.37)	– 0.296 (0.51)	– 0.31 (0.49)	0.57	0.35	Gaussian (link = identity)		

Dummy coding is used and reference categories are not shown

*Time(2)* follow-up measure after 2 months, *Group(nicu)* COVID patients that did not needed intensive care, $\sigma_{pat}^{2}$ variance component for patients variability, $\sigma_{res}^{2}$ residual variance, *SE* standard error, *ICC* intraclass correlation coefficient, *BIC* Bayesian Information Criteria.

*p < 0.05, **p < 0.01, ***p < 0.001

#### Characteristic path length

A multilevel model with a Gaussian distribution and log-link function is used. Characteristic path length decreased significantly over time (exp(*b*) = 0.97, *p* = 0.006). No significant effects at the group level (exp(*b*) = 1.07, *p* = 0.442) nor significant interaction effects (exp(*b*) = 1.02, *p* = 0.220) were found. The analyses showed an ICC of 0.76, meaning that 76% of the variation in the outcome variable was accounted for by the clustering structure of the data. Next, a sensitivity analysis was performed to make sure that the results cannot solely be attributed to two outlying cases with missing values at follow-up. Therefore, the analysis was redone but without these two observations and similar results were found.

**Fig. 2:**
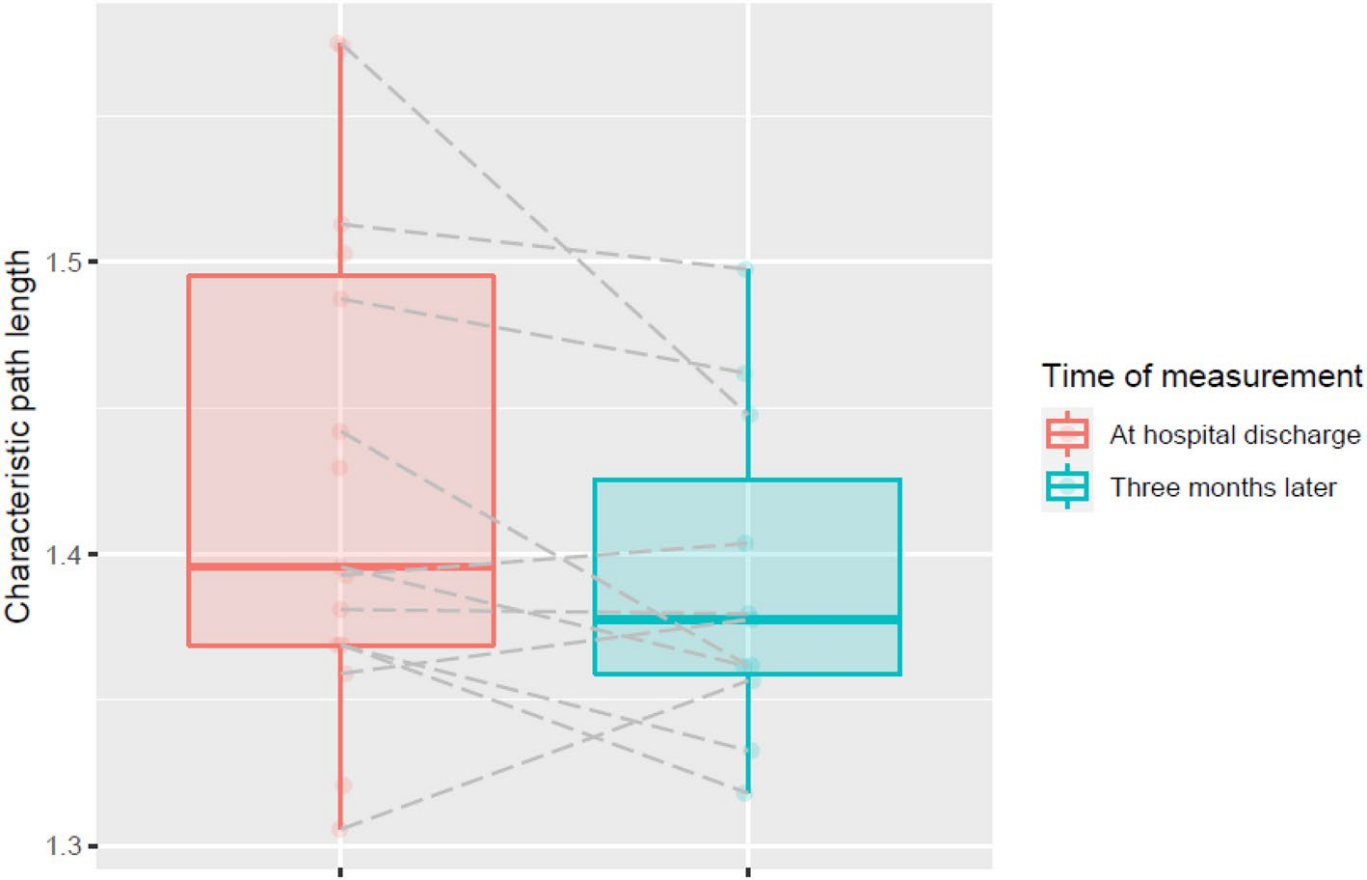
Characteristic path length: significant effect of time (*p* = 0.01) remains after sensitivity analysis. Represented are the median and the IQR (box), Q1 − 1.5 × IQR and Q3 + 1.5 × IQR (whiskers), and individual observations (dots)

Because no significant effect of group was found. Figure 1 can be simplified to Fig. 2.

#### Cluster coefficient, global efficiency, and local efficiency

Statistical analyses revealed no significant main effect of time, of group nor interaction effects for these outcome measures.

### Cognitive performance

**Fig. 3:**
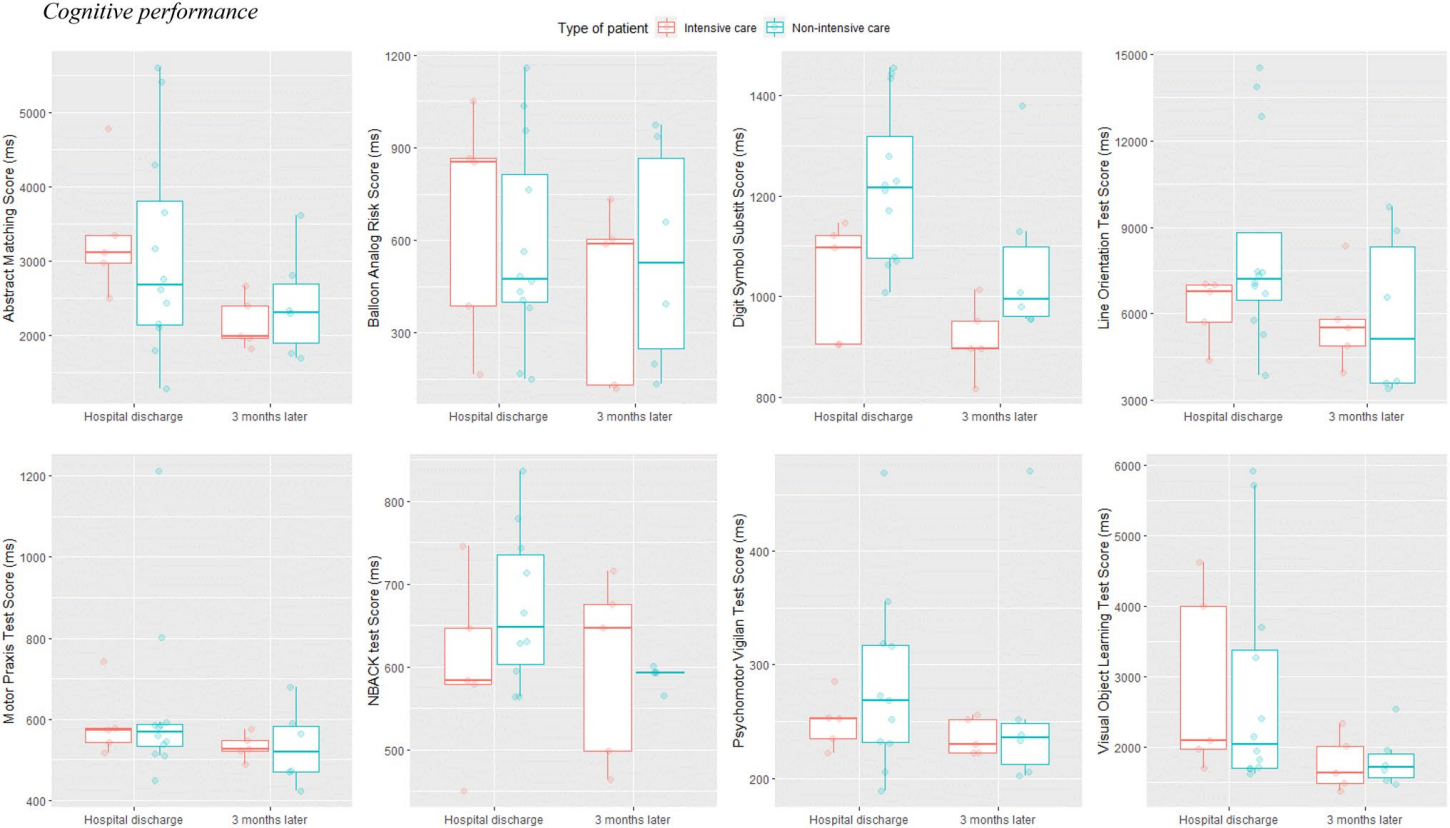
Overview of median reaction time in ICU (red) and non-ICU (blue) patients over a 3 month time period after hospital discharge across all eight cognitive tests

Figure 3 and Table 4 present the median reaction times over a 3 month time period after hospital discharge across all cognitive tests. Using (univariate) multilevel models (see Table 5 for more information), a significant effect of time (p < 0.05) was found for the Abstract Matching, Balloon Analogue Risk Test, Digit Symbol Substitution Test, and Psychomotor Vigilance Test. Both non-ICU and ICU COVID-19 survivors improved their cognitive performance on these four cognitive tests 2 months later. For the Digit Symbol Substitution Test, a significant group effect was found (*p* = 0.01) with the ICU group performing better than the non-ICU group. No significant effects were observed for the Visual Object Learning Test, Motor Praxis Test, Line Orientation Test, and NBACK.

**Table 4:** Raw median reaction time (ms) data of all cognitive performance tests

Cognitive test	ICU		Non-ICU	
	At discharge	At 3 months	At discharge	At 3 months
Abstract Matching	3346 ± 862	2171 ± 351	3109 ± 1379	2422 ± 714
Balloon Analogue Risk Test	665 ± 371	435 ± 329	581 ± 328	549 ± 364
Digit Symbol Substitution Test	1035 ± 120	914 ± 73	1222 ± 157	1068 ± 166
Line Orientation Test	6175 ± 1135	5707 ± 1647	8263 ± 3494	5972 ± 2838
Motor Praxis Test	590 ± 88	532 ± 104	622 ± 192	533 ± 95
NBACK	596 ± 96	549 ± 70	624 ± 98	587 ± 96
Psychomotor Vigilance Test	250 ± 23	236 ± 17	282 ± 80	267 ± 56
Visual Object Learning Test	2878 ± 1332	1772 ± 399	2805 ± 1548	1828 ± 391

Values are expressed as mean ± standard deviation

**Table 5:** Additional information on the cognitive performance analyses

Cognitive test	Final model	BIC	ICC	Estimate	Standard error	Significance level
Abstract matching test	Multilevel model with a gaussian distribution and identity link function	428.77	0.61	Time: − 1175.24	447.61	**0.041**
				Group: − 237.65	599.43	0.70
				Interaction: 552.48	592.32	0.38
Balloon analogue risk test	Linear mixed model	–	–	Time: − 229.42	95.61	**0.040**
				Group: − 84.38	178.34	0.64
				Interaction: 184.81	127.96	0.18
Digit symbol substitution test	Linear mixed model	–	–	Time: − 119.84	40.49	**0.016**
				Group: 187.37	77.24	**0.027**
				Interaction: − 15.41	54.22	0.78
Visual object learning test	Multilevel model with a gaussian distribution and identity link function	434.82	–	Time: − 1105.38	576.64	0.094
				Group: − 72.76	650.31	0.91
				Interaction: 168.71	755.66	0.83
Psychomotor vigilance test	Multilevel model with a gamma distribution and a log-link function	260.63	0.83	Time: − 0.053	0.025	**0.034**
				Group: 0.091	0.16	0.56
				Interaction: 0.08	0.036	**0.021**
Motor praxis test	Multilevel model with a gaussian distribution and identity link function	335.81	–	Time: − 58.70	87.74	0.52
				Group: 31.99	79.65	0.69
				Interaction: − 35.29	112.85	0.76
Line orientation test	Multilevel model with a gaussian distribution and identity link function	470.22	–	Time: − 467.86	860.49	0.60
				Group: 2087.85	1561.94	0.20
				Interaction: − 1545.47	1150.80	0.21
NBACK	Multilevel model with a gaussian distribution (and identity link function)	272.87	–	Time: − 0.0027	0.038	0.94
				Group: 0.12	0.10	0.24
				Interaction: − 0.074	0.056	0.19

The bold values indicate significant results and are provided in this way to detect the significant effects easily

*BIC*  Bayesian information criterion, *ICC* intraclass correlation coefficient

### Association between structural brain connectivity and cognitive performance

A step-wise model-building technique was applied to investigate the association between structural brain connectivity and cognitive performance. Table 6 presents the Akaike Information Criterion (AIC) as an estimate of the quality of each model, as well as the corresponding p values and adjusted R-squared values for each cognitive task.

**Table 6:** Final models including AIC, adjusted R-squares, and *P* values

Outcome	Final model	Coefficient (SE)	AIC	Adjusted R-squared
Digit symbol substitution test	Intercept	1063.12 (58.55)***	168.54	0.41
	Group(nicu)	162.17 (71.02)*		
	CC	– 638.20 (266.29)*		
	CP	357.05 (187.74) +		
Visual object learning test	Intercept	2623.95 (330.37)***	245.81	0.19
	CC	5337.91 (2477.12)*		
Motor praxis test	Intercept	617.55 (39.87)***	175.28	0.13
	LE	97.30 (53.59) +		
NBACK	Intercept	6.46 (0.04)***	183.53	
	CP	0.29 (0.19)		
Psychomotor vigilance test	Intercept	5.61 (0.06)***	180.31	
Balloon analogue risk test	Intercept	605.28 (80.47)***	198.32	
Abstract matching test	Intercept	3151.89 (287.82)***	242.49	0.07
	LE	– 570.99 (386.80)		
Line orientation test	Intercept	7441.81 (749.03)***	274.38	0.05
	CP	5405.32 (3911.83)		

Dummy coding is used and reference categories are not shown

*Group(nicu)* COVID patients that did not needed intensive care, *CP* characteristic path length, *LE* local efficiency, *CC* cluster coefficient, *SE* standard error, *AIC* Akaike Information Criteria.

^+^*p* < 0.10, *p < 0.05, **p < 0.01, ***p < 0.001

**Fig. 4:**
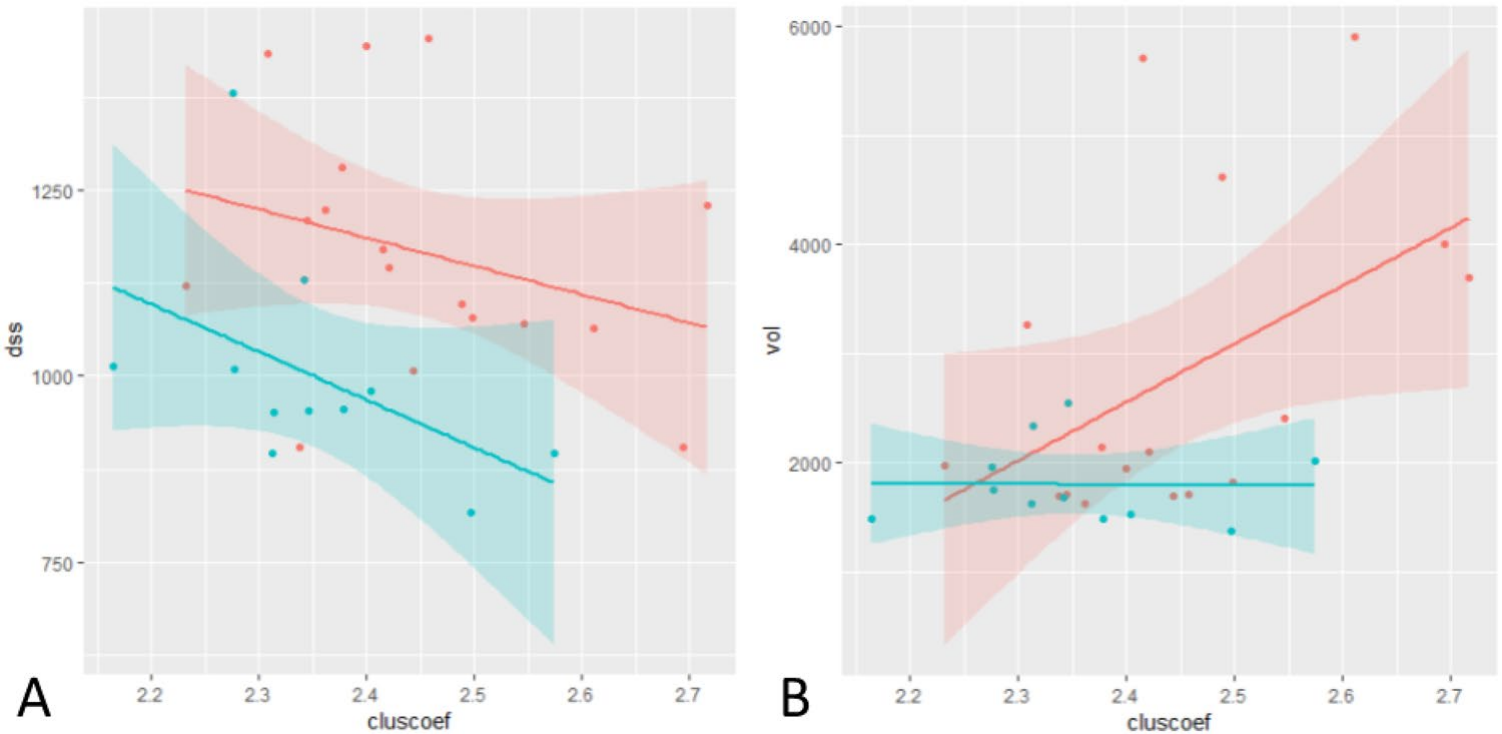
Association between scores on the Digit Symbol Substitution Task and Cluster coefficient (**A**) and on the Visual Object Learning Task and cluster coefficient (**B**) in COVID-19 survivors at time of hospital discharge (time 1 in red) and 2 months later (time 2 in blue). An lm smoother is shown, together with its 95% confidence interval. Dots represent individual observations

A significant association was observed between the Digit Symbol Substitution Test and cluster coefficient (*p* = 0.018; adjusted *R*^2^ = 0.41) and between the Visual Object Learning Test and cluster coefficient (*p* = 0.048) (Fig. 4).

No significant association was found between any of the graph metrics and cognitive performance on the Motor Praxis Test, NBACK, Psychomotor Vigilance Test, Balloon Analogue Risk Test, Abstract Matching Test, and Line Orientation Test.

## Discussion

The aim of the current study was to assess structural brain connectivity in non-ICU- and ICU-treated COVID-19 survivors up to 2 months after discharge, as well as cognitive performance by means of a standardized cognitive test battery assessing different cognitive domains. Results over time showed a decrease in characteristic path length, indicating an increased potential for information transmission (Parhizi et al. 2018; Zhang et al. 2020). Also, cognitive performance in terms of reaction time on the Abstract Matching Test, Balloon Analogue Risk Test, Digit Symbol Substitution Test, and Psychomotor Vigilance Test improved over time. The structural brain connectivity measures did not associate well with the cognitive performance measures.

The overall results of this study are encouraging, as former non-ICU and ICU COVID-19 patients showed modest improvements in both cognitive performance and characteristic path length 2 months after hospital discharge. Also, no deterioration in structural brain connectivity, cognitive performance, and brain structure outcomes was observed in these former hospitalized COVID-19 patients after 2 months of hospital discharge. Combined, these results might suggest early signs of neurological and cognitive recovery in formerly admitted COVID-19 patients 2 months after hospital discharge.

A previous MRI-based follow-up study of COVID-19 patients found an overall decrease in DTI values (i.e., mean diffusivity, axial diffusivity, and radial diffusivity) in combination with an increase in fractional anisotropy at the 3-month interval compared with non-COVID-19 participants. It was observed that the global mean diffusivity among others was correlated with memory loss (Lu et al. 2020). On the contrary, Yang et al (2021) used DTI, and observed that COVID-19 patients exhibiting decreased fractional anisotropy, increased mean diffusivity, and radial diffusivity values in widespread brain regions, as well as significantly lower global efficiency, longer characteristic path length, and less nodal local efficiency in the superior occipital gyrus (Yang et al. 2021). Both studies (9,18) also reported that the altered white matter changes are unfavorable when comparing recovered COVID-19 patients to healthy controls. In the context of these findings, our results suggest a shortening of characteristic path length over time, which indicates better information transmission 2 months after hospital discharge and could infer recovery of underlying neurological functioning (Parhizi et al. 2018; Zhang et al. 2020). Nevertheless, a more rigorous study design is warranted to substantiate this claim as well as to align results of previous DTI studies.

Expanding on the cognitive performance aspect in our study, patients’ cognitive performance improved on four out of seven cognitive tests over time (i.e., Abstract Matching Test, Balloon Analogue Risk Test, Digit Symbol Substitution Test, and Psychomotor Vigilance Test). The Abstract Matching Test measures the individual’s ability to group stimuli in a logical way (abstraction) and to learn undisclosed rules based on feedback. Tasks assessing abstraction and concept formation activate the prefrontal cortex primarily (Berman et al. 1995). The Balloon Analogue Risk Test assesses risk decision-making behavior (Lejuez et al. 2002; Rao et al. 2008). It has been shown to consistently activate the orbital frontal and ventromedial prefrontal cortex, amygdala, hippocampus, anterior cingulate cortex, and ventral striatum (Basner et al. 2015). The Digit Symbol Substitution Test assesses complex scanning and visual tracking. This test requires a combination of visual scanning and eye–hand coordination, where the temporal cortex, prefrontal cortex, and motor cortex are primarily involved in this process (Basner et al. 2015). The Psychomotor Vigilance Test assesses vigilant attention by measuring the reaction time of how fast the patient can respond to the onset of a millisecond counter (Basner and Dinges 2011). The brain regions associated with this cognitive test and domain are the prefrontal cortex, motor cortex, inferior parietal, and visual cortex. In summary, both ICU and non-ICU COVID-19 survivors showed an improvement in these specific cognitive domains over 2 months. Future studies with a greater sample size should explore the specific brain areas associated with these four cognitive tests.

The interpretation of our study findings is in line with previous research reporting that COVID-19-related persistent symptoms improved over time, even though patient reported neurocognitive symptoms can persist for 12 months and more after acute COVID-19 infection in a considerable subgroup of these patients (Kim et al. 2022). While the results of our study suggest slight improvements in structural brain connectivity and cognitive performance in previously hospitalized COVID-19 patients after 2 months, deficiencies in cognitive functioning are still present at 1, 3 and 6 months compared to healthy controls (Poletti et al. 2021). Six months after the COVID-19 infection, cognitive function assessed by the Montreal Cognitive Assessment was worse in formerly infected people who were not hospitalized compared to people who were never infected by the virus (Del Brutto et al. 2021a). At 1-year follow-up, the COVID-19 survivors’ improved their cognitive function and no differences remained with cognitive functioning of the non-infected people (Del Brutto et al. 2021a). These results were partially confirmed in a population of COVID-19 patients who were formerly hospitalized. The formerly hospitalized patients did not show a worse cognitive function compared to healthy controls at the 1-year follow-up moment, but more white matter abnormalities were still observed in comparison to healthy controls (Huang et al. 2021). In general, it seems that cognitive function and performance are likely to recover in formerly infected COVID-19 patients with presentations from mild symptoms to have required intensive hospital care, though the underlying brain structure and function might need longer to recover.

One of the novelties of this paper is the exploration of the link between structural brain connectivity and cognitive performance. Using a step-wise model-building technique, we observed associations between the Digit Symbol Substitution Test, the Visual Object Learning Test, and the cluster coefficient (Table 6 and Fig. 4). The cluster coefficient represents the ratio of links between the chosen node and the nearest-neighboring nodes and the number of possible links (Onias et al. 2014). This could mean that individuals with a higher cluster coefficient have a lower reaction time and thus have a better cognitive performance. Figure 4 shows that the reaction time on both cognitive tasks decreases over time and the cluster coefficient increases, highlighting that the adverse effects after a COVID-19 infection are reversible. No further associations were observed between shorter characteristic path lengths and faster reaction times, probably due to the small sample size. In the future, more emphasis might be placed on the specific brain area responsible for improved cognitive functioning, in this case, the ventral tegmental area.

No further associations were observed between shorter characteristic path length and faster reaction times, probably due to the small sample size. In the future, more emphasis might be set at the specific brain area responsible for improved cognitive functioning, in this case, the ventral tegmental area.

Therefore, besides the small sample size, the main limitations of this study are the lack of a healthy matched control group and the prevalence of comorbidities in our sample (e.g., diabetes = 35%, overweight and obesity = 70%, arterial hypertension = 25%, and dyslipidaemia = 20%). Despite encouraging exploratory results in structural brain connectivity and cognitive performance during the relative short follow-up period (2 months) of COVID-19 survivors after hospital discharge, future studies should include more participants and a healthy matched control group and take into account comorbidities (MacIntosh et al. 2021; Moheet et al. 2015).

Also, we mainly focused on cortical cognitive functioning, and did not test whether and how more subcortical affective processing such for instance emotion reactivity and emotion regulation might be influenced. Moreover, future studies should also incorporate frequent and longer follow-up periods, and target specific subpopulations experiencing persistent COVID-19 symptoms as up to more than 20% of the patients still report concentration difficulties, cognitive dysfunction, and amnesia 12 months after infection (Kim et al. 2022).

## Conclusion

Two months after hospital discharge, former hospitalized non-ICU- and ICU-treated COVID-19 survivors showed an improvement of structural brain connectivity, indicated by a decreased characteristic path length. Also, performance on different cognitive tasks indicated by a faster reaction time improved over time. Detrimental effects of COVID-19 on brain function and structure ameliorate over time. Future scope should include longer follow-up, aim at unraveling the impact of COVID-19 on the subcortical affective processing and investigate the associations between structural brain connectivity and cognitive performance.

## Data Availability

The data that support the findings of this study are available on request from the corresponding author. The data are not publicly available due to privacy or ethical restrictions.

## References

[CR1] Avants BB, Tustison NJ, Song G, Cook PA, Klein A, Gee JC (2011). A reproducible evaluation of ANTs similarity metric performance in brain image registration. Neuroimage.

[CR2] Bahranifard B, Mehdizadeh S, Hamidi A, Khosravi A, Emami R, Mirzaei K, Nemati R, Nemati F, Assadi M, Gholamrezanezhad A (2021). A review of neuroradiological abnormalities in patients with coronavirus disease 2019 (COVID-19). Neuroradiol J.

[CR3] Basner M, Dinges DF (2011). Maximizing sensitivity of the psychomotor vigilance test (PVT) to sleep loss. Sleep.

[CR4] Basner M, Savitt A, Moore TM, Port AM, McGuire S, Ecker AJ, Nasrini J, Mollicone DJ, Mott CM, McCann T, Dinges DF, Gur RC (2015). Development and validation of the cognition test battery for spaceflight. Aerosp Med Human Perform.

[CR5] Bastiani M, Cottaar M, Fitzgibbon SP, Suri S, Alfaro-Almagro F, Sotiropoulos SN, Jbabdi S, Andersson JLR (2019). Automated quality control for within and between studies diffusion MRI data using a non-parametric framework for movement and distortion correction. Neuroimage.

[CR6] Bates D, Mächler M, Bolker B, Walker S (2015). Fitting linear mixed-effects models using lme4. J Stat Softw.

[CR7] Berman KF, Ostrem JL, Randolph C, Gold J, Goldberg TE, Coppola R, Carson RE, Herscovitch P, Weinberger DR (1995). Physiological activation of a cortical network during performance of the Wisconsin Card Sorting Test: a positron emission tomography study. Neuropsychologia.

[CR8] Blazhenets G, Schroeter N, Bormann T, Thurow J, Wagner D, Frings L, Weiller C, Meyer PT, Dressing A, Hosp JA (2021). Slow but evident recovery from neocortical dysfunction and cognitive impairment in a series of chronic COVID-19 patients. J Nucl Med.

[CR9] Cothran TP, Kellman S, Singh S, Beck JS, Powell KJ, Bolton CJ, Tam JW (2020). A brewing storm: The neuropsychological sequelae of hyperinflammation due to COVID-19. Brain Behav Immun.

[CR10] de Erausquin GA, Snyder H, Carrillo M, Hosseini AA, Brugha TS, Seshadri S (2021). The chronic neuropsychiatric sequelae of COVID-19: the need for a prospective study of viral impact on brain functioning. Alzheimers Dement.

[CR11] Del Brutto OH, Rumbea DA, Recalde BY, Mera RM (2021). Cognitive sequelae of long COVID may not be permanent: a prospective study. Eur J Neurol.

[CR12] Del Brutto OH, Wu S, Mera RM, Costa AF, Recalde BY, Issa NP (2021). Cognitive decline among individuals with history of mild symptomatic SARS-CoV-2 infection: a longitudinal prospective study nested to a population cohort. Eur J Neurol.

[CR13] Desikan RS, Ségonne F, Fischl B, Quinn BT, Dickerson BC, Blacker D, Buckner RL, Dale AM, Maguire RP, Hyman BT, Albert MS, Killiany RJ (2006). An automated labeling system for subdividing the human cerebral cortex on MRI scans into gyral based regions of interest. Neuroimage.

[CR14] Douaud G, Lee S, Alfaro-Almagro F, Arthofer C, Wang C, McCarthy P, Lange F, Andersson JLR, Griffanti L, Duff E, Jbabdi S, Taschler B, Keating P, Winkler AM, Collins R, Matthews PM, Allen N, Miller KL, Nichols TE, Smith SM (2022). SARS-CoV-2 is associated with changes in brain structure in UK Biobank. Nature.

[CR15] Duong D (2021). Even mild COVID-19 may have long-term brain impacts. CMAJ.

[CR16] European Centre for Disease Prevention and Control (2022). COVID-19 [Online]. https://www.ecdc.europa.eu/en/geographical-distribution-2019-ncov-cases. Accessed 1 Feb 2022

[CR17] Fischl B (2012). FreeSurfer. Neuroimage.

[CR18] Fotuhi M, Mian A, Meysami S, Raji CA (2020). Neurobiology of COVID-19. J Alzheimer's Dis JAD.

[CR19] Frontera JA, Sabadia S, Lalchan R, Fang T, Flusty B, Millar-Vernetti P, Snyder T, Berger S, Yang D, Granger A, Morgan N, Patel P, Gutman J, Melmed K, Agarwal S, Bokhari M, Andino A, Valdes E, Omari M, Kvernland A, Lillemoe K, Chou SH, McNett M, Helbok R, Mainali S, Fink EL, Robertson C, Schober M, Suarez JI, Ziai W, Menon D, Friedman D, Friedman D, Holmes M, Huang J, Thawani S, Howard J, Abou-Fayssal N, Krieger P, Lewis A, Lord AS, Zhou T, Kahn DE, Czeisler BM, Torres J, Yaghi S, Ishida K, Scher E, de Havenon A, Placantonakis D, Liu M, Wisniewski T, Troxel AB, Balcer L, Galetta S (2021). A prospective study of neurologic disorders in hospitalized patients with COVID-19 in New York City. Neurology.

[CR20] Frontera JA, Yang D, Lewis A, Patel P, Medicherla C, Arena V, Fang T, Andino A, Snyder T, Madhavan M, Gratch D, Fuchs B, Dessy A, Canizares M, Jauregui R, Thomas B, Bauman K, Olivera A, Bhagat D, Sonson M, Park G, Stainman R, Sunwoo B, Talmasov D, Tamimi M, Zhu Y, Rosenthal J, Dygert L, Ristic M, Ishii H, Valdes E, Omari M, Gurin L, Huang J, Czeisler BM, Kahn DE, Zhou T, Lin J, Lord AS, Melmed K, Meropol S, Troxel AB, Petkova E, Wisniewski T, Balcer L, Morrison C, Yaghi S, Galetta S (2021). A prospective study of long-term outcomes among hospitalized COVID-19 patients with and without neurological complications. J Neurol Sci.

[CR21] Gorbalenya AE, Baker SC, Baric RS, de Groot RJ, Drosten C, Gulyaeva AA, Haagmans BL, Lauber C, Leontovich AM, Neuman BW, Penzar D, Perlman S, Poon LLM, Samborskiy DV, Sidorov IA, Sola I, Ziebuhr J, Coronaviridae Study Group of the International Committee on Taxonomy of, V (2020). The species Severe acute respiratory syndrome-related coronavirus: classifying 2019-nCoV and naming it SARS-CoV-2. Nat Microbiol.

[CR22] Hampshire A, Trender W, Chamberlain SR, Jolly AE, Grant JE, Patrick F, Mazibuko N, Williams SC, Barnby JM, Hellyer P, Mehta MA (2021) Cognitive deficits in people who have recovered from COVID-19. EClinicalMedicine 39:101044 10.1016/j.eclinm.2021.101044 PMC8298139 34316551

[CR23] Hopkins RO, Jackson JC (2006). Long-term Neurocognitive Function After Critical Illness. Chest.

[CR24] Hosp JA, Dressing A, Blazhenets G, Bormann T, Rau A, Schwabenland M, Thurow J, Wagner D, Waller C, Niesen WD, Frings L, Urbach H, Prinz M, Weiller C, Schroeter N, Meyer PT (2021). Cognitive impairment and altered cerebral glucose metabolism in the subacute stage of COVID-19. Brain.

[CR25] Huang S, Zhou Z, Yang D, Zhao W, Zeng M, Xie X, Du Y, Jiang Y, Zhou X, Yang W, Guo H, Sun H, Liu P, Liu J, Luo H, Liu J (2021). Persistent white matter changes in recovered COVID-19 patients at the 1-year follow-up. Brain.

[CR26] Iadecola C, Anrather J, Kamel H (2020). Effects of COVID-19 on the nervous system. Cell.

[CR27] Jain S, Sima DM, Ribbens A, Cambron M, Maertens A, van Hecke W, de Mey J, Barkhof F, Steenwijk MD, Daams M, Maes F, van Huffel S, Vrenken H, Smeets D (2015). Automatic segmentation and volumetry of multiple sclerosis brain lesions from MR images. Neuroimage Clin.

[CR28] Jenkinson M, Beckmann CF, Behrens TEJ, Woolrich MW, Smith SM (2012). FSL. Neuroimage.

[CR29] Jorge Cardoso M, Leung K, Modat M, Keihaninejad S, Cash D, Barnes J, Fox NC, Ourselin S (2013). STEPS: similarity and truth estimation for propagated segmentations and its application to hippocampal segmentation and brain parcelation. Med Image Anal.

[CR30] Kanberg N, Simrén J, Edén A, Andersson LM, Nilsson S, Ashton NJ, Sundvall PD, Nellgård B, Blennow K, Zetterberg H, Gisslén M (2021). Neurochemical signs of astrocytic and neuronal injury in acute COVID-19 normalizes during long-term follow-up. EBioMedicine.

[CR31] Kim Y, Bitna H, Kim S-W, Chang H-H, Kwon KT, Bae S, Hwang S (2022). Post-acute COVID-19 syndrome in patients after 12 months from COVID-19 infection in Korea. BMC Infect Dis.

[CR33] Kremer S, Lersy F, Anheim M, Merdji H, Schenck M, Oesterlé H, Bolognini F, Messie J, Khalil A, Gaudemer A, Carré S, Alleg M, Lecocq C, Schmitt E, Anxionnat R, Zhu F, Jager L, Nesser P, Mba YT, Hmeydia G, Benzakoun J, Oppenheim C, Ferré JC, Maamar A, Carsin-Nicol B, Comby PO, Ricolfi F, Thouant P, Boutet C, Fabre X, Forestier G, de Beaurepaire I, Bornet G, Desal H, Boulouis G, Berge J, Kazémi A, Pyatigorskaya N, Lecler A, Saleme S, Edjlali-Goujon M, Kerleroux B, Constans JM, Zorn PE, Mathieu M, Baloglu S, Ardellier FD, Willaume T, Brisset JC, Caillard S, Collange O, Mertes PM, Schneider F, Fafi-Kremer S, Ohana M, Meziani F, Meyer N, Helms J, Cotton F (2020). Neurologic and neuroimaging findings in patients with COVID-19: A retrospective multicenter study. Neurology.

[CR34] Kremer S, Lersy F, De Sèze J, FerrÉ J-C, Maamar A, Carsin-Nicol B, Collange O, Bonneville F, Adam G, Martin-Blondel G, Rafiq M, Geeraerts T, Delamarre L, Grand S, Krainik A, Caillard S, Constans JM, Metanbou S, Heintz A, Helms J, Schenck M, Lefèbvre N, Boutet C, Fabre X, Forestier G, De Beaurepaire I, Bornet G, Lacalm A, Oesterlé H, Bolognini F, Messié J, Hmeydia G, Benzakoun J, Oppenheim C, Bapst B, Megdiche I, Henry Feugeas M-C, Khalil A, Gaudemer A, Jager L, Nesser P, Talla Mba Y, Hemmert C, Feuerstein P, Sebag N, Carré S, Alleg M, Lecocq C, Schmitt E, Anxionnat R, Zhu F, Comby P-O, Ricolfi F, Thouant P, Desal H, Boulouis G, Berge J, Kazémi A, Pyatigorskaya N, Lecler A, Saleme S, Edjlali-Goujon M, Kerleroux B, Zorn P-E, Matthieu M, Baloglu S, Ardellier F-D, Willaume T, Brisset JC, Boulay C, Mutschler V, Hansmann Y, Mertes P-M, Schneider F, Fafi Kremer S, Ohana M, Meziani F, David JS, Meyer N, Anheim M, Cotton F (2020). Brain MRI findings in severe COVID-19: a retrospective observational Study. Radiology.

[CR35] Kuznetsova A, Brockhoff PB, Christensen RHB (2017). lmerTest package: tests in linear mixed effects models. J Stat Softw.

[CR36] Lapucci C, Romano N, Schiavi S, Saitta L, Uccelli A, Boffa G, Pardini M, Signori A, Castellan L, Inglese M, Roccatagliata L (2020). Degree of microstructural changes within T1-SE versus T1-GE hypointense lesions in multiple sclerosis: relevance for the definition of “black holes”. Eur Radiol.

[CR37] Lejuez CW, Read JP, Kahler CW, Richards JB, Ramsey SE, Stuart GL, Strong DR, Brown RA (2002). Evaluation of a behavioral measure of risk taking: the Balloon Analogue Risk Task (BART). J Exp Psychol Appl.

[CR38] Lersy F, Willaume T, Brisset JC, Collange O, Helms J, Schneider F, Chammas A, Willaume A, Meyer N, Anheim M, Cotton F, Kremer S (2021). Critical illness-associated cerebral microbleeds for patients with severe COVID-19: etiologic hypotheses. J Neurol.

[CR39] Liguori C, Pierantozzi M, Spanetta M, Sarmati L, Cesta N, Iannetta M, Ora J, Mina GG, Puxeddu E, Balbi O, Pezzuto G, Magrini A, Rogliani P, Andreoni M, Mercuri NB (2020). Subjective neurological symptoms frequently occur in patients with SARS-CoV2 infection. Brain Behav Immun.

[CR40] Lu Y, Li X, Geng D, Mei N, Wu PY, Huang CC, Jia T, Zhao Y, Wang D, Xiao A, Yin B (2020). Cerebral micro-structural changes in COVID-19 patients—an MRI-based 3-month follow-up study. EClinicalMedicine.

[CR41] Luigetti M, Iorio R, Bentivoglio AR, Tricoli L, Riso V, Marotta J, Piano C, Primiano G, Zileri Del Verme L, Lo Monaco MR, Calabresi P (2020). Assessment of neurological manifestations in hospitalized patients with COVID-19. Eur J Neurol.

[CR42] Macintosh BJ, Ji X, Chen JJ, Gilboa A, Roudaia E, Sekuler AB, Gao F, Chad JA, Jegatheesan A, Masellis M, Goubran M, Rabin J, Lam B, Cheng I, Fowler R, Heyn C, Black SE, Graham SJ (2021). Brain structure and function in people recovering from COVID-19 after hospital discharge or self-isolation: a longitudinal observational study protocol. CMAJ Open.

[CR43] Mao L, Jin H, Wang M, Hu Y, Chen S, He Q, Chang J, Hong C, Zhou Y, Wang D, Miao X, Li Y, Hu B (2020). Neurologic manifestations of hospitalized patients with coronavirus disease 2019 in Wuhan, China. JAMA Neurol.

[CR44] Mazziotta JC, Toga AW, Evans A, Fox P, Lancaster J (1995). A probabilistic atlas of the human brain: theory and rationale for its development. The International Consortium for Brain Mapping (ICBM). Neuroimage.

[CR45] Meppiel E, Peiffer-Smadja N, Maury A, Bekri I, Delorme C, Desestret V, Gorza L, Hautecloque-Raysz G, Landre S, Lannuzel A, Moulin S, Perrin P, Petitgas P, Sella IF, Wang A, Tattevin P, de Broucker T (2021). Neurologic manifestations associated with COVID-19: a multicentre registry. Clin Microbiol Infect.

[CR46] Moheet A, Mangia S, Seaquist ER (2015). Impact of diabetes on cognitive function and brain structure. Ann N Y Acad Sci.

[CR47] Montalvan V, Lee J, Bueso T, de Toledo J, Rivas K (2020). Neurological manifestations of COVID-19 and other coronavirus infections: a systematic review. Clin Neurol Neurosurg.

[CR48] Onias H, Viol A, Palhano-Fontes F, Andrade KC, Sturzbecher M, Viswanathan G, de Araujo DB (2014). Brain complex network analysis by means of resting state fMRI and graph analysis: Will it be helpful in clinical epilepsy?. Epilepsy Behav.

[CR49] Parhizi B, Daliri MR, Behroozi M (2018). Decoding the different states of visual attention using functional and effective connectivity features in fMRI data. Cogn Neurodyn.

[CR50] Poletti S, Palladini M, Mazza MG, De Lorenzo R, Furlan R, Ciceri F, Rovere-Querini P, Benedetti F (2021). Long-term consequences of COVID-19 on cognitive functioning up to 6 months after discharge: role of depression and impact on quality of life. Eur Arch Psychiatry Clin Neurosci.

[CR51] R Core Team (2021) R: a language and environment for statistical computing [Online]. Vienna, Austria: R Foundation for Statistical Computing. https://www.R-project.org/ Accessed

[CR52] Rao H, Korczykowski M, Pluta J, Hoang A, Detre JA (2008). Neural correlates of voluntary and involuntary risk taking in the human brain: an fMRI Study of the Balloon Analog Risk Task (BART). Neuroimage.

[CR53] Schilling KG, Blaber J, Huo Y, Newton A, Hansen C, Nath V, Shafer AT, Williams O, Resnick SM, Rogers B, Anderson AW, Landman BA (2019). Synthesized b0 for diffusion distortion correction (Synb0-DisCo). Magn Reson Imaging.

[CR54] Smeets D, Ribbens A, Sima DM, Cambron M, Horakova D, Jain S, Maertens A, van Vlierberghe E, Terzopoulos V, van Binst AM, Vaneckova M, Krasensky J, Uher T, Seidl Z, de Keyser J, Nagels G, de Mey J, Havrdova E, van Hecke W (2016). Reliable measurements of brain atrophy in individual patients with multiple sclerosis. Brain Behav.

[CR55] Smith RE, Tournier J-D, Calamante F, Connelly A (2012). Anatomically-constrained tractography: Improved diffusion MRI streamlines tractography through effective use of anatomical information. Neuroimage.

[CR56] Smith RE, Tournier J-D, Calamante F, Connelly A (2013). SIFT: Spherical-deconvolution informed filtering of tractograms. Neuroimage.

[CR57] Smith RE, Tournier J-D, Calamante F, Connelly A (2015). SIFT2: Enabling dense quantitative assessment of brain white matter connectivity using streamlines tractography. Neuroimage.

[CR58] Song E, Zhang C, Israelow B, Lu-Culligan A, Prado AV, Skriabine S, Lu P, Weizman O-E, Liu F, Dai Y, Szigeti-Buck K, Yasumoto Y, Wang G, Castaldi C, Heltke J, Ng E, Wheeler J, Alfajaro MM, Levavasseur E, Fontes B, Ravindra NG, van Dijk D, Mane S, Gunel M, Ring A, Kazmi SAJ, Zhang K, Wilen CB, Horvath TL, Plu I, Haik S, Thomas J-L, Louvi A, Farhadian SF, Huttner A, Seilhean D, Renier N, Bilguvar K, Iwasaki A (2021). Neuroinvasion of SARS-CoV-2 in human and mouse brain. J Exp Med.

[CR59] Sunaert S, Radwan A (2021) KULeuven Neuro Imaging Suite [Online]. Github. https://github.com/treanus/KUL_NIS. Accessed 2021

[CR60] Tasker RC, Menon DK (2016). Critical care and the brain. JAMA.

[CR61] Tournier J-D, Calamante F, Connelly A (2010) Improved probabilistic streamlines tractography by 2nd order integration over fibre orientation distributions. In: Proc. Intl. Soc. Mag. Reson. Med. (ISMRM), p 18

[CR62] Tournier J-D, Smith R, Raffelt D, Tabbara R, Dhollander T, Pietsch M, Christiaens D, Jeurissen B, Yeh C-H, Connelly A (2019). MRtrix3: a fast, flexible and open software framework for medical image processing and visualisation. BioRxiv.

[CR63] Tournier JD, Calamante F, Connelly A (2007). Robust determination of the fibre orientation distribution in diffusion MRI: Non-negativity constrained super-resolved spherical deconvolution. Neuroimage.

[CR64] Venables W, Ripley B (2002). Modern applied statistics with S.

[CR65] Wang Y, Ghumare E, Vandenberghe R, Dupont P (2017). Comparison of different generalizations of clustering coefficient and local efficiency for weighted undirected graphs. Neural Comput.

[CR66] Yachou Y, El Idrissi A, Belapasov V, Ait Benali S (2020). Neuroinvasion, neurotropic, and neuroinflammatory events of SARS-CoV-2: understanding the neurological manifestations in COVID-19 patients. Neurol Sci.

[CR67] Yang L, Zhou M, Li L, Luo P, Fan W, Xu J, Chen Q, Pan F, Lei P, Zheng C, Jin Y (2021). Characteristics of mental health implications and plasma metabolomics in patients recently recovered from COVID-19. Transl Psychiatry.

[CR68] Zhang W, Guo L, Liu D, Xu G (2020). The dynamic properties of a brain network during working memory based on the algorithm of cross-frequency coupling. Cogn Neurodyn.

